# A Spaceborne Integrated S/Ka Dual-Band Dual-Reflector Antenna

**DOI:** 10.3390/mi17010124

**Published:** 2026-01-18

**Authors:** Zenan Yang, Weiqiang Han, Liang Tang, Haihua Wang, Yilin Wang, Yongchang Jiao

**Affiliations:** 1 School of Electronic Engineering, Xidian University, Xi’an 710071, China; 2 Shanghai Aerospace Electronic Technology Institute, Shanghai 201109, China; hanweiqiangsky@163.com; 3 Shanghai Aerospace System Engineering Institute, Shanghai 201109, China; tl_ht805@163.com; 4 Xi’an Leitong Technology Co., Ltd., Xi’an 710075, China; kingseaflower@163.com (H.W.); wangyilinchn@163.com (Y.W.)

**Keywords:** satellite communication, frequency-selective surface, dual-band antenna, dual-reflector antenna

## Abstract

To address the diverse requirements of satellite communication applications involving medium-/low-rate reliable links and high-rate high-capacity services, an integrated S/Ka dual-band dual-reflector antenna is proposed as an effective solution. Owing to the stringent spatial constraints of satellite platforms, the longer operating wavelengths in the S-band lead to oversized feed horns in the integrated antenna design, which induces severe secondary aperture blockage, thus degrading aperture efficiency and impeding practical mechanical layout implementation. To alleviate this critical drawback, the proposed antenna achieves multi-band aperture reuse by deploying an array with four miniaturized S-band radiating elements around a broadband Ka-band feed horn. A frequency-selective surface (FSS)-based sub-reflector is further designed to effectively enhance the effective aperture size for the S-band operation, while ensuring unobstructed electromagnetic propagation in the Ka-band, thus enabling simultaneous dual-band high-gain radiation. After comprehensive electromagnetic simulation and parametric optimization for the antenna feed and the FSS sub-reflector, experimental measurements verify that the S-band left-hand and right-hand circularly polarized (LHCP/RHCP) channels achieve more than 20.2 dBic gains with more than 6° half-power beamwidths (HPBWs), and the Ka-band channel yields gains exceeding 41.2 dBic, with HPBWs greater than 0.8°.

## 1. Introduction

From the perspective of service coverage in satellite communication, the S-band, owing to its low rain attenuation characteristic (with significantly lower path loss than the Ka-band under heavy rain conditions), serves as a critical backbone for medium-to-low-rate reliable communication scenarios, such as emergency command. However, traditional S-band helical antennas suffer from low gains, failing to meet high-speed service demands. By leveraging its ultra-wide bandwidth advantage, the Ka-band enables high-speed data transmission, making it a viable choice for high-throughput satellites and satellite internet applications requiring large capacity. Neither band alone can cover the full scenario spectrum encompassing “reliable low-to-medium-rate + high-speed large-capacity.” From an engineering implementation perspective, traditional discrete S/Ka antennas are significantly larger and heavier than integrated designs, conflicting with the lightweighting trend of spaceborne platforms. They also require additional resources to mitigate inter-band interference. In contrast, dual-band integrated designs unify both frequency band characteristics into a single architecture. This not only achieves full-service coverage but also overcomes the bottlenecks of discrete designs in terms of size, weight, and interference control. Consequently, the necessity of dual-band antennas has become an industry consensus in satellite communication.

Reflector antennas offer advantages such as high aperture efficiency, compact structure, and high reliability, thus being widely applied in satellite communication, navigation, and relay applications. Based on satellite service requirements, an integrated S/Ka dual-band antenna for spaceborne communication is designed using a reflector antenna configuration. A reflector antenna consists of a feed horn and a reflector, where the feed horn is the critical component determining the reflector’s efficiency. Currently, research on multi-band feed horns primarily focuses on two categories, i.e., coaxial horn feeds [1,2,3,4,5] and multi-feed array designs [6]. In the first category, multi-band coverage is achieved by nesting horns for different frequency bands in a coaxial structure. These feeds feature a compact architecture and high efficiency, but suffer from complex feed networks, stringent layout requirements, heavier weight, and severe secondary aperture blockage at lower frequencies. While in the second category, multiple feeds are employed to cover multiple bands. Advantages of multi-feed array designs include simplified feed networks and compact layouts, while their disadvantages include the inability of multiple feeds to achieve focal point alignment, leading to reduced efficiency in specific bands. Additionally, miniaturization of feeds at lower operating frequencies compromises the radiation cone, resulting in reduced efficiency of the reflector antenna.

To meet the dual-frequency operation requirements of satellites in the S-band and Ka-band, this paper presents a ring-focus dual-reflector antenna configuration integrated with a spatial envelope layout design. In the S-band feed, a miniaturized folded-element array design utilizing a four-element radiating array is employed to enhance the illumination cone and improve S-band efficiency. While in the Ka-band feed, an axial-wave corrugated horn design is adopted to enhance the stability of the high-frequency phase center. Adding an FSS to the edge of the sub-reflector improves S-band efficiency without affecting Ka-band performance. Experimental results validate that the proposed antenna satisfies the satellite communication requirements for both the S-band and Ka-band.

## 2. Antenna Requirements

In accordance with the satellite communication requirements for the S/Ka-band frequencies, gains of the S-band antenna should be greater than 19 dBic (accounting for feed network losses), with more than 6° HPBWs, dual circular polarization (DCP) capability, and less than 3 dB axial ratios (ARs). For the Ka-band antenna, greater than 39 dBic gains (accounting for feed network losses), more than 0.8° HPBWs, the LHCP operation, and less than 3 dB ARs are required. Detailed performance specifications are summarized in Table 1.

## 3. Reflector Antenna Design

### 3.1. Overall Reflector Design

Owing to the stringent spatial constraints that preclude the accommodation of two independent reflectors, an integrated design strategy is employed for development of the S/Ka dual-band composite antenna. Given the stringent longitudinal spatial envelope constraints on the satellite-mounted antennas, a single-reflector configuration, which exhibits a relatively large longitudinal footprint, fails to meet the layout requirements. Consequently, in this paper, a dual-reflector antenna design featuring a more compact longitudinal envelope is adopted. Currently, the dual-reflector antenna topologies widely utilized in aerospace applications include the Cassegrain and ring-focus configurations [7]. The sub-reflector of a ring-focus dual-reflector antenna employs an elliptical surface profile. Electromagnetic waves emitted by the feed horn are reflected by the sub-reflector, with the strongest electric field focused on the edge of the primary reflector. In contrast, the electric field reflected back to the feed horn is relatively weak, resulting in excellent VSWR performance and high aperture efficiency [8]. Thus, a ring-focus dual-reflector configuration is selected for the proposed antenna.

Per the aperture antenna theory, the antenna gain is calculated by Equation (1) [9].

(1) $$G=\frac{4\pi A_{e}}{\lambda^{2}}\eta$$

The empirical formula for the antenna’s HPBW is provided in Equation (2) [10].

(2) $$\theta_{3\mathrm{dB}}=67.64\frac{\lambda }{D}$$

where

- G: gain of the dual-reflector antenna;
- $A_{e}$: effective aperture area of the reflector antenna;
- λ: operating wavelength;
- η: aperture efficiency of the antenna;
- $\theta_{3\mathrm{dB}}$: HPBW of the reflector antenna;
- D: aperture diameter of the reflector.

Per Equation (1), with a reflector aperture diameter of 1000 mm (equivalent to an effective area of 1 m^2^) and an aperture efficiency of 60%, an on-axis gain of 43.8 dBic can be achieved in the Ka-band. Per Equation (2), the calculated 3 dB HPBW of the antenna in Ka-band is 1.03°. In the system design, a 1 dB loss budget is reserved for the Ka-band feed network. Based on the aforementioned calculations, the proposed antenna satisfies the requirement of gains greater than 39 dBic with HPBWs greater than 0.8°. For the S-band, in contrast, the longer operating wavelengths necessitate specialized design considerations to meet aperture efficiency requirements.

### 3.2. Composite Feed Design

To improve product reliability, a commercially available mature reflector product with a focal length-to-diameter ratio of f/D = 0.27 is employed, where f denotes the focal length and D is the aperture diameter of the reflector. According to these calculations, the total height of the feed horn (including the feed network) should be less than 196 mm, which poses significant challenges to the design of the S/Ka composite feed system. In this section, a multi-feed integrated design strategy is adopted for the development of the S/Ka composite feed system. For the Ka-band, a corrugated horn antenna with a stable phase center is employed as the radiating feed element. The preliminary estimation formula for the feed horn aperture radius is given by Equation (3) [11].

(3) $$\{\begin{array}{c} f_{\mathrm{cTE}11}=\frac{87.849}{R}\\ f_{\mathrm{cTM}01}=\frac{114.743}{R} \end{array}$$

In Equation (3), $f_{\mathrm{cTE}11}$ represents the cutoff frequency of the TE_11_ mode within the circular waveguide, $f_{\mathrm{cTM}01}$ denotes the cutoff frequency of the TM_01_ mode, and R is the aperture radius of the circular waveguide. During the design of the circular waveguide horn antenna, the selection of the circular waveguide radius must ensure the efficient transmission of the TE_11_ mode while suppressing the TM_01_ mode. Per Equation (3), the aperture radius of the axial-wave corrugated horn antenna is determined as R = 6 mm to achieve the objectives of minimizing the lateral dimensions and reducing its impact on the inter-element spacing of the array. A three-layer corrugated structure is adopted for the axial corrugations, with specific parameters optimized via electromagnetic simulation software.

Owing to the longer operating wavelengths in the S-band, a single antenna element is unable to meet the required illumination taper. Consequently, a four-element array design is adopted in this paper to meet the illumination taper requirements. Constrained by the internal space of the reflector, miniaturization of the S-band antenna array is essential. This miniaturization not only accommodates spatial constraints but also reduces feed blockage, thereby improving the aperture efficiency of the reflector antenna. Monopole antennas are selected as the array elements, and folding technologies are employed for miniaturization. To adapt to the satellite communication environment, the antenna ground plane and the support cylinder are integrated into a single structure. The antenna elements are mounted on the support cylinder using polyimide dielectric sheets, which enhances the mechanical robustness of the array and improves the overall reliability of the product.

**Figure 1:**
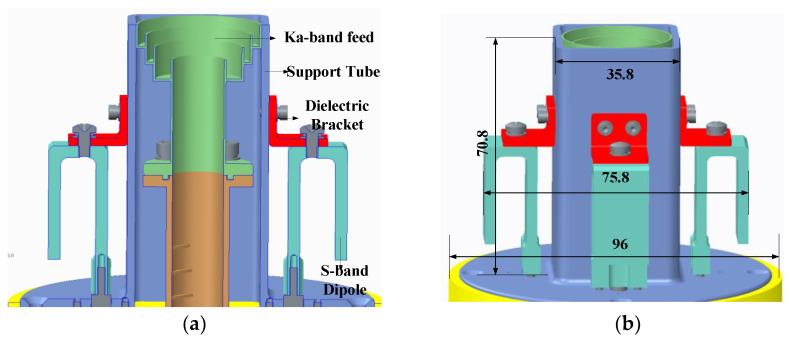
Structure of feed: (**a**) cross-sectional view; (**b**) overall view.

**Figure 2:**
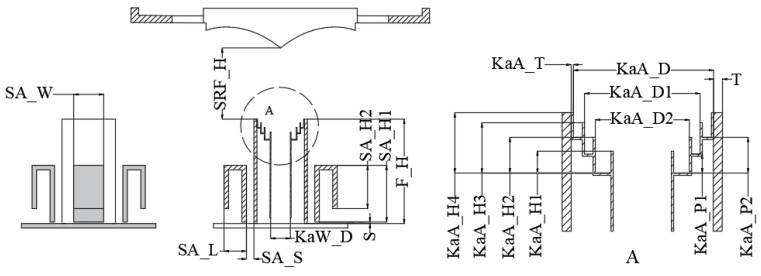
Schematic diagram of key parameters for the overall simulation.

The Ka-band feed horn is integrated within the support cylinder of the S-band feed system, forming a composite feed system through a unified design approach. This composite feed system is positioned at the focal point of the reflector to enhance the radiation efficiency of both the S-band and Ka-band. The external configuration of the S/Ka composite feed system is illustrated in Figure 1, which labels the overall dimensions in millimeters (mm), while the specific dimensional parameters of the feed system are provided in the subsequent Figure 2 and Table 2.

**Figure 3:**
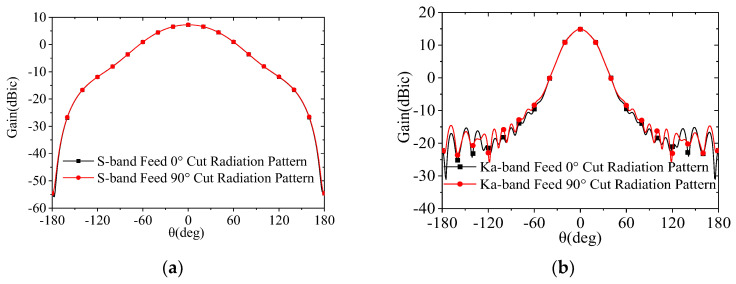
Simulated radiation patterns of the feed: (**a**) S-band; (**b**) Ka-band.

Through optimization via electromagnetic simulation software, the maximum overall dimensions of the composite feed system are determined as Φ75.8 mm. The radiation patterns of the S-band and Ka-band feed horns are presented in Figure 3. As shown in the figure, the Ka-band feed horn achieves an illumination taper of −14 dB, meeting the design requirements, with excellent pattern uniformity across all the planes and an amplitude variation of less than 0.1 dB within the illumination angles. In contrast, the S-band feed system exhibits an illumination taper of approximately −5 dB, resulting in relatively lower efficiency. The solution to this issue will be specifically addressed in Section 3.4.

### 3.3. Feed Network Design

Primary types of Ka-band waveguide circular polarizers include dielectric polarizers [12], septum polarizers [13], and corrugated polarizers [14]. Corrugated polarizers exhibit distinct advantages such as low insertion loss, wide operating bandwidth, and a compact structure. Consequently, in this design, we employ a compact corrugated polarizer featuring low insertion loss and favorable manufacturability for practical implementation.

The corrugated polarizer operates primarily in the TE_11_ mode, and its polarization conversion mechanism is elaborated as follows. When the incident polarized wave impinges on the corrugations at a 45° angle, it decomposes into two orthogonal linearly polarized components—one perpendicular to the corrugations and the other parallel to them. For the component parallel to the corrugations, the corrugated structure acts as a shunt inductance; for the component perpendicular to the corrugations, it acts as a shunt capacitance. This discrepancy in electrical properties induces a phase lag of the perpendicularly polarized component relative to the parallel component. By optimizing the geometric parameters of the corrugated section, a 90° phase difference between these two orthogonal components can be achieved at the output port, ultimately generating circularly polarized radiation. Furthermore, by adjusting the incident angle of the polarized wave relative to the corrugations, the polarization mode can be switched. A 45° incident angle corresponds to the LHCP, while a 135° incident angle corresponds to the RHCP, thus meeting the requirements of diverse application scenarios.

**Figure 4:**
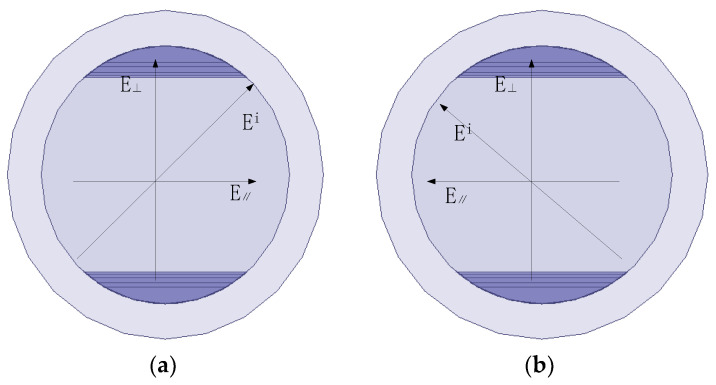
Schematic of the polarizer: (**a**) LHCP; (**b**) RHCP.

The core performance of this circular polarizer is determined by the insertion depth, spacing, and thickness of the corrugated diaphragms within the corrugated section. These parameters directly influence the polarization conversion precision and impedance matching performance. Currently, the mainstream corrugated diaphragm distribution profiles include the raised-cosine distribution and the Gaussian function distribution. Considering the structural simplicity and manufacturability, the raised-cosine distribution scheme is adopted in this design. Its three-dimensional (3D) model and operational principle are illustrated in Figure 4.

**Figure 5:**
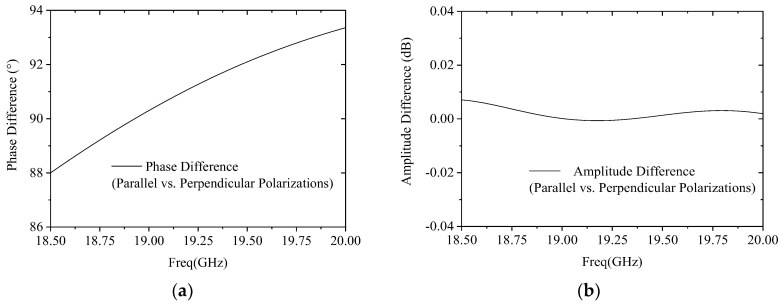
Simulated results of the polarizer: (**a**) phase difference; (**b**) amplitude difference.

Figure 5 presents the simulation validation results of this design. Within the operating frequency band, the phase differences between these two orthogonal polarized waves deviate from the theoretical 90° phase values by only approximately 2.5°, and the amplitude fluctuation is constrained within 0.01 dB. The corresponding ARs remain consistently below 0.2 dB, fully verifying the effectiveness and reliability of the proposed design.

**Figure 6:**
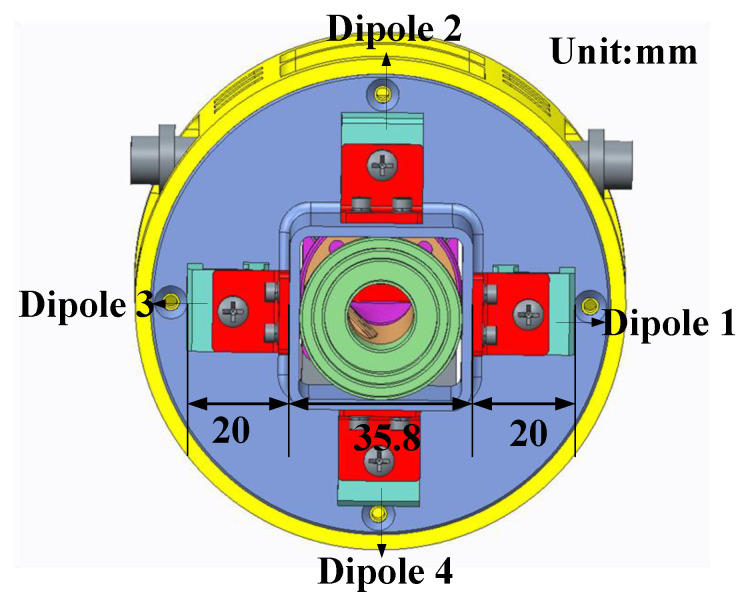
S-band array layout.

The DCP operation for the S-band is achieved via a four-element antenna array configuration. The four antenna elements are arranged in the layout illustrated in Figure 6. Notably, the realization of circular polarization relies on a specific phase excitation configuration among these four elements. With Element 1 designated as the phase reference, the correspondence between the polarization modes and the element phase settings is elaborated in Table 3.

**Figure 7:**
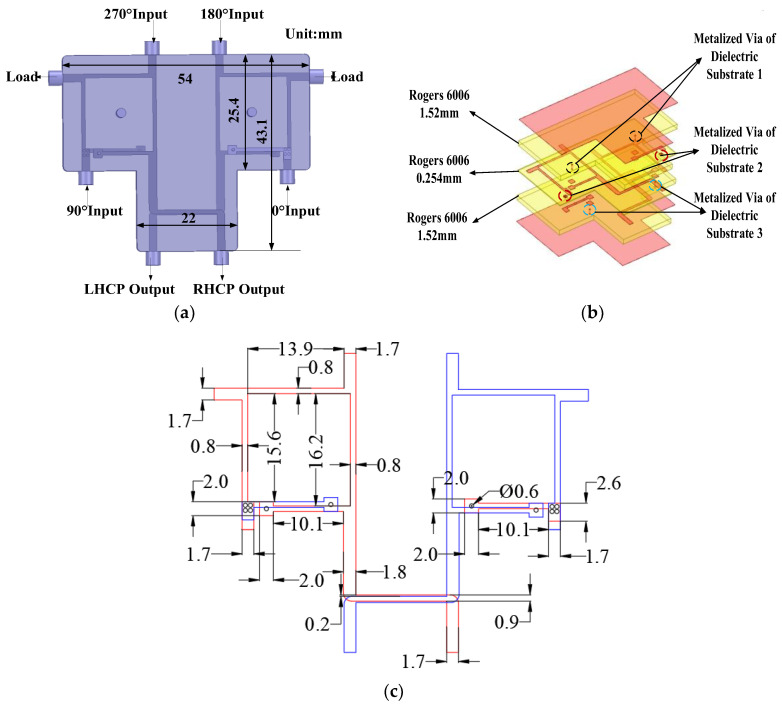
The S-band polarizer. (**a**) Device Geometry; (**b**) 3D view of dielectric substrate stack; (**c**) planar dimensions of critical structures (Unit: mm).

According to the phase relationship of the antenna elements presented in Table 2, the required phase differences among the four elements can be achieved via two network schemes. The first scheme utilizes one 90° hybrid coupler and two 180° hybrid couplers; the second scheme employs one 180° hybrid coupler and two 90° hybrid couplers. Consequently, the first scheme is adopted in this design. Notably, constrained by the limited space available for the antenna layout, the conventional approach employing three independent networks results in issues including excessive spatial occupation, complex interconnection cable routing, and additional insertion loss. To address this challenge, a compact, integrated DCP network is designed to enable a more space-efficient layout. As illustrated in Figure 7, the S-band polarizer comprises three double-sided copper-clad dielectric substrates, secured with screws. The main circuits are patterned on the top and bottom copper layers of the second substrate; symmetry between these layers enables derivation of the device’s overall dimensions from the dimensions of a single layer.

**Figure 8:**
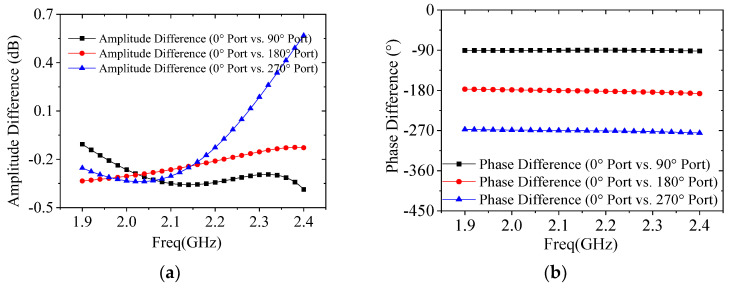
Simulated results of the S-band polarizer: (**a**) amplitude difference; (**b**) phase difference.

Figure 8 presents the simulation validation results for the S-band DCP performance. Within the operating frequency band, the simulated phase differences between these two orthogonal polarized waves deviate by approximately 4.3° from the theoretical values, with an amplitude fluctuation of around 0.4 dB. The corresponding ARs are approximately 2 dB, which meets the design requirements.

### 3.4. Frequency-Selective Surface Sub-Reflector Design

As elaborated in Section 3.2, the S-band feed employs a miniaturized design and a multi-element array topology. However, its illumination taper is only −5 dB, thus failing to meet the design specifications. Two conventional approaches for addressing this issue are inherently constrained. First, increasing the feed dimensions can enhance taper performance, but this is restricted by the limited internal space of the reflector and would lead to excessive blockage of the sub-reflector, making this solution infeasible. Second, enlarging the sub-reflector dimensions can reduce S-band spillover and improve taper performance, yet this would significantly degrade Ka-band radiation efficiency, introducing a performance trade-off between these two frequency bands.

To mitigate this trade-off, an optimized solution, i.e., integrating an FSS around the periphery of the sub-reflector, is proposed. By utilizing the frequency-selective characteristic of the FSS, the enlarged sub-reflector is designed to exhibit high reflectivity for the S-band and high transmissivity for the Ka-band. This effectively increases the effective aperture area of the sub-reflector for the S-band, thereby improving its taper performance while avoiding interference with the Ka-band radiation characteristics. Consequently, synergistic performance optimization for both frequency bands is achieved.

An FSS is a type of frequency-selective electromagnetic metamaterial. Its core functionality is realized through precise periodic structural design, enabling the passband characteristics for the preset target frequencies and the stopband characteristics for the non-target frequencies [15]. Typical existing FSS unit structures include the periodic arrays of circular apertures, circular patches, ring structures, and Jerusalem crosses [16,17,18]. Notably, the periodic circular aperture structure offers distinct advantages, such as a simplified geometry, well-established fabrication technology, and compatibility with conformal design via flat-plate perforation. These advantages are well-aligned with the stringent product reliability requirements in the aerospace domain. Consequently, the periodic circular aperture structure is selected for integration with the sub-reflector in this design.

**Figure 9:**
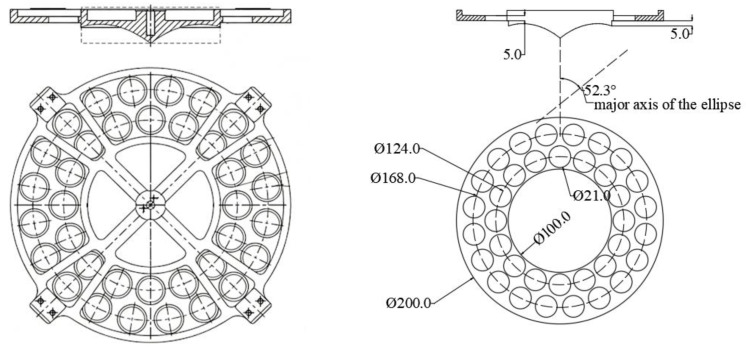
Structure and main dimensions of the sub-reflector (unit: mm).

To further ensure the mechanical reliability of the product in the space environment, the reinforcing ribs are designed and integrated on the backside of the sub-reflector. The specific configuration of the developed FSS sub-reflector is illustrated in Figure 9. Its detailed structural composition is depicted as follows. The central region is the ring-focus sub-reflector, which employs an elliptical surface of revolution to match the electromagnetic characteristics of the main reflector. Starting from a position 5 mm above the edge of the elliptical surface, a circular ring surface extends radially outward. The inner diameter of this ring surface matches the outer diameter of the elliptical surface, and its outer diameter is designed to be twice the inner diameter. Two rows of periodic circular apertures are fabricated in this ring surface area—this perforated ring constitutes the functional FSS layer. Its electromagnetic characteristic design objective is to achieve total reflection for the S-band and total transmission for the Ka-band. Figure 9 further lists the primary dimensional parameters for the numerical simulation, and the elliptical surface has a major axis of 49.35 mm and a minor axis of 37.89 mm.

**Figure 10:**
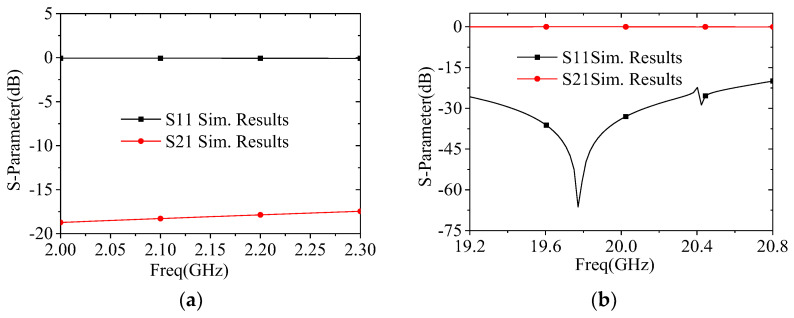
Simulated results of the FSS: (**a**) S-band; (**b**) Ka-band.

Figure 10 presents the simulated S parameters of the FSS for the S-band and Ka-band. Analyses of the simulation data reveal the following results. Within the S-band operating range, the reflection coefficient (S_11_) of the FSS is close to 0 dB, meeting the total reflection design requirement. Within the Ka-band operating range, the reflection coefficient (S_11_) remains stably below −20 dB, and the transmission loss (S_12_) averages approximately 0.1 dB. The total transmission performance fully meets the specified requirements, verifying the effectiveness of the proposed FSS design.

### 3.5. Overall Optimization of Reflector Design

**Figure 11:**
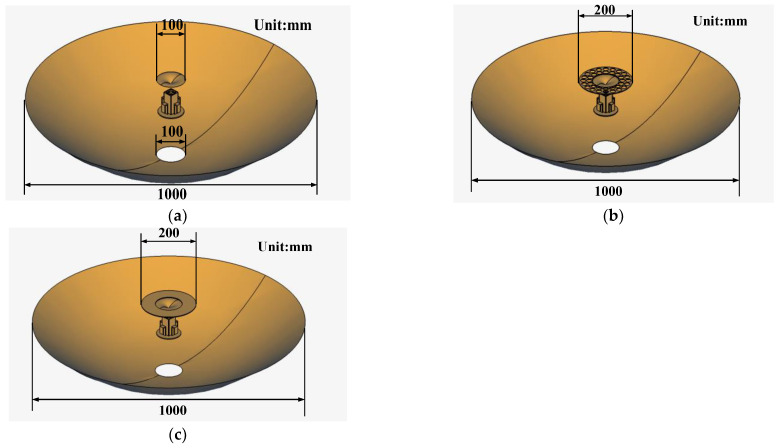
Three simulation models of the antenna: (**a**) without FSS; (**b**) with FSS; (**c**) enlarged sub-reflector.

**Figure 12:**
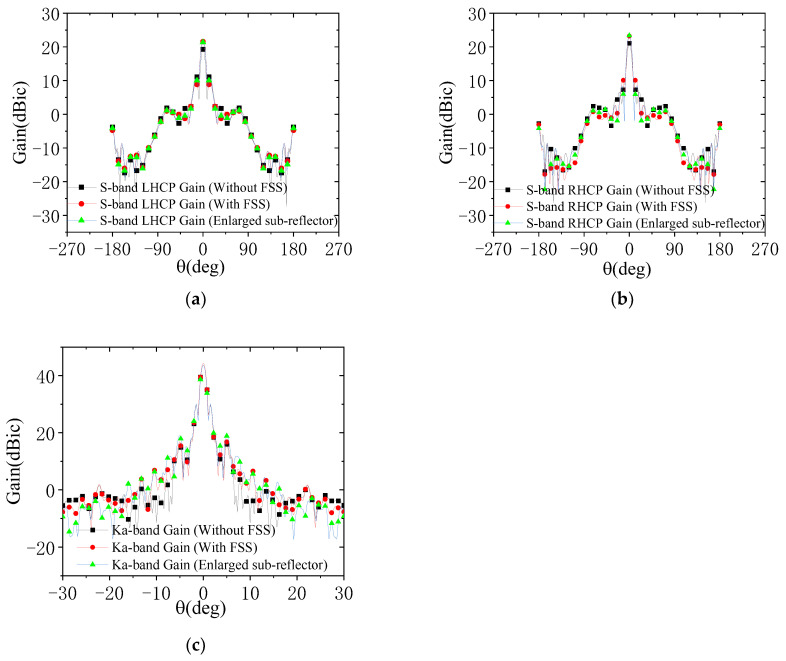
Simulated radiation patterns of three models: (**a**) S-band LHCP; (**b**) S-band RHCP; (**c**) K-band.

To clarify the effects of FSS integration and sub-reflector enlargement on dual-reflector antenna performance, comprehensive full-link simulations are performed. Three configurations, i.e., the FSS-integrated sub-reflector, enlarged sub-reflector, and original sub-reflector, are compared. Electromagnetic simulation models of these three configurations are shown in Figure 11. In the simulation models, dimensions of the main reflector and aperture are referred to in Section 3.1 and Section 3.4, respectively, and other key dimensions are shown in Figure 2 and Table 2. As shown in Figure 12, both FSS integration and sub-reflector enlargement enhance S-band gains. Notably, FSS integration improves S-band gain without affecting Ka-band performance, while sub-reflector enlargement degrades Ka-band gains.

Table 4, which shows the simulated gains excluding feed network losses, confirms that within the specified beamwidth, the S-band on-axis and beam-edge gains increase for both the FSS-integrated and enlarged sub-reflector configurations. The key conclusion is that FSS integration boosts S-band gains without impacting Ka-band performance, whereas the mere sub-reflector enlargement impairs Ka-band gains.

## 4. Experimental Verification

**Figure 13:**
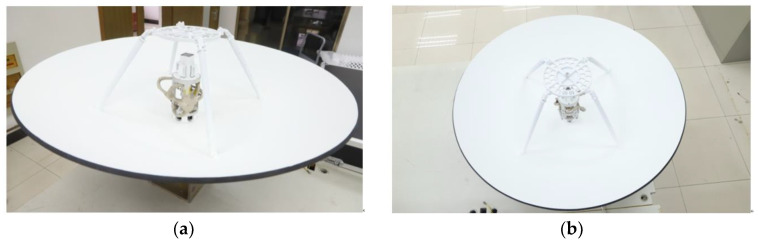
Photographs of the assembled prototype: (**a**) side view; (**b**) top view.

To validate the antenna performance, an antenna prototype is fabricated and assembled. The assembled prototype comprises a reflector antenna, an integrated composite feed, an S-band DCP network, and a Ka-band circular polarizer. The reflector has an aperture of 1 m, and the internal space of the feed support cylinder is constrained. The miniaturized design of the S-band DCP network facilitates its installation within the feed support cylinder. Photographs of the assembled reflector antenna are provided in Figure 13.

**Figure 14:**
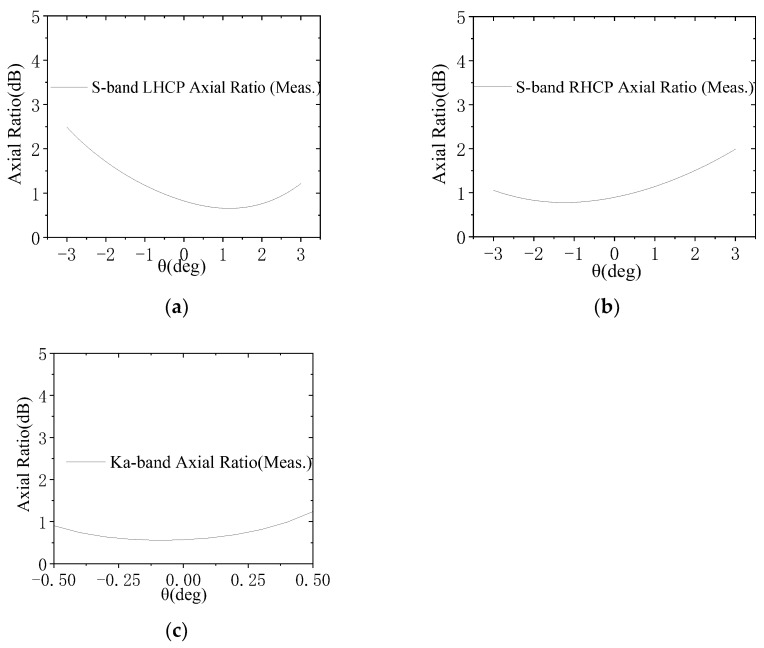
Measured AR patterns of the assembled prototype: (**a**) S-band LHCP; (**b**) S-band RHCP; (**c**) Ka-band.

**Figure 15:**
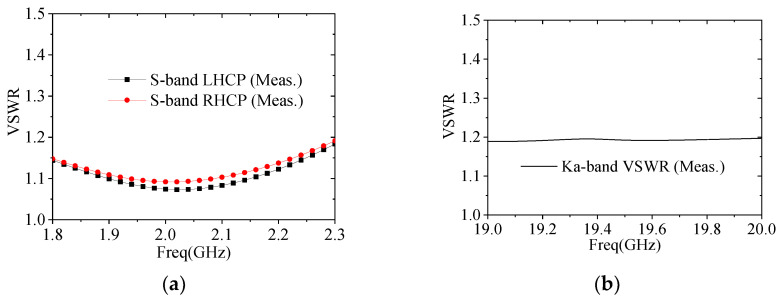
VSWRs of the assembled prototype: (**a**) S-band; (**b**) Ka-band.

**Figure 16:**
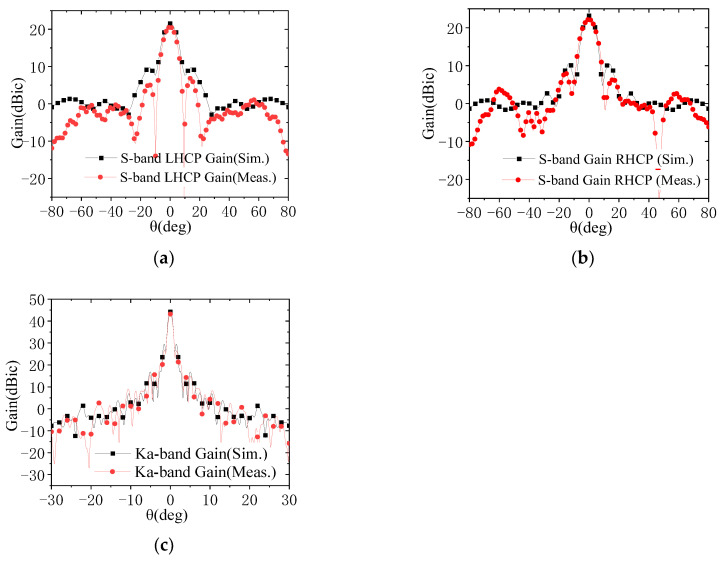
Measured and simulated radiation patterns of the assembled prototype: (**a**) S-band LHCP; (**b**) S-band RHCP; (**c**) Ka-band.

The assembled reflector antenna is subjected to radiation characteristic testing in a planar near-field range. The measured AR patterns are illustrated in Figure 14. As shown in this figure, within the 6° observation beamwidths, the measured ARs for both the S-band RHCP and LHCP channels are less than 2.5 dB. For the Ka-band, the measured ARs are less than 1.3 dB within the 0.8° observation beamwidths. The measured VSWRs are presented in Figure 15, with the VSWRs of each antenna channel remaining below 1.2. Figure 16 presents the measured gain values for both frequency bands, with the simulated results also included for comparison. Notably, the testing encompasses the circular polarizers and their associated interconnecting components, including cables and waveguides. As shown in Figure 16, the on-beam gains of the S-band channel are greater than 20.2 dBic, while those of the Ka-band channel are greater than 41.2 dBic, with both frequency bands exhibiting a performance margin of over 2 dB. Since the actual measured beamwidths are very close to the simulated ones, the gain reductions (compared to the simulated values) may be attributed to backend feeder losses not considered in the simulation and test errors. Nevertheless, the overall performance meets the design requirements. A comparison between the measured radio frequency (RF) performance of the antenna and the specification requirements is provided in Table 5.

## 5. Conclusions

This paper presents the design methodology for a spaceborne S/Ka dual-band integrated antenna. Electromagnetic simulation software is employed to optimize and design its key components, including the composite feed, circular polarizers, and FSS, as well as the overall antenna system. Both simulation and experimental measurement results demonstrate that the antenna achieves excellent electromagnetic performance. Specifically, for the S-band, both the LHCP and RHCP channels yield gains greater than 20.2 dBic with the ARs below 2.5 dB within the 6° observation beamwidths; for the Ka-band, the operating channel yields gains greater than 41.2 dBic with the ARs below 1.3 dB within the 0.8° observation beamwidths.

The key innovation of integrating an FSS ring on the periphery of the conventional sub-reflector effectively addresses the long-standing challenge of low radiation efficiency in the low-frequency band for small-aperture reflector antennas under stringent spatial constraints. This design solution significantly improves radiation efficiencies in the S-band while fully preserving high radiation efficiencies in the Ka-band, and this proposed approach exhibits substantial application potential for future small satellite platforms.

From a structural perspective, structural modification is minimal and highly reliable—consisting essentially of an enlarged sub-reflector area with periodic apertures—thus maintaining the inherently high reliability of reflector antenna systems. Furthermore, this design strategy provides a valuable technical reference for improving efficiencies in individual frequency bands for multi-band reflector antenna systems.

## Figures and Tables

**Table 1:** Specification requirements of antenna.

Parameters	S-Band Channel Specifications	Ka-Band Channel Specifications
Axial gain/dBic	≥19	≥39
HPBW/°	≥6	≥0.8
Polarization	DCP	LHCP
Axial ratio/dB	≤3	≤3
VSWR ^1^	≤1.5	≤1.5
Aperture	≤1 m	≤1 m

^1^ VSWR: voltage standing wave ratio.

**Table 3:** Phase configuration requirements for the S-band array elements.

Port	RHCP	LHCP
Phase of Element 1/°	0	0
Phase of Element 2/°	−90	90
Phase of Element 3/°	−180	180
Phase of Element 4/°	−270	270

**Table 4:** Comparison of simulation results for the three models.

Parameters	Without FSS			With FSS			Enlarged Sub-Reflector		
	S-Band LHCP	S-Band RHCP	Ka-Band	S-Band LHCP	S-Band RHCP	Ka-Band	S-Band LHCP	S-Band RHCP	Ka-Band
Axial Gain/dBic	≥19.3	≥21	≥44.2	≥21.6	≥23.5	≥44.3	≥21.4	≥23.4	≥43.6
Observation Beamwidth/°	6		0.8	6		0.8	6		0.8
On-beam Gain/dBic	≥17.6	≥19.4	≥42.2	≥20.3	≥21.6	≥42.3	≥20.1	≥21.8	≥41.6

**Table 2:** List of parameter values.

Parameters	KaA_D	KaA_D1	KaA_D2	KaW_D	KaA_H1	KaA_H2	KaA_H3	KaA_H4	Ka_P1	Ka_P2	Ka_T
Values/mm	31.8	25.8	21	13	4.9	8	11.25	13.5	4.25	8	0.5
Parameters	SA_L	SA_H1	SA_H2	SA_S	SA_W	SA_T	S	F_H	SRF_H	T	
Values/mm	15	38	29	5	16	3	1	70.8	47.8	2	

**Table 5:** Statistical results of radio frequency performance of the assembled prototype.

Parameters	S-Band		Ka-Band	
	Specification	Measurement	Specification	Measurement
Axial gain/dBic	≥18.5	≥20.2	≥39	≥41.2
HPBW/°	≥6	≥6	≥0.8	≥0.8
Polarization	DCP	DCP	LHCP	LHCP
Axial ratio/dB	≤3	≤2.5	≤3	≤1.3
VSWR	≤1.5	≤1.19	≤1.5	≤1.18
Aperture	1 m		1 m	

## Data Availability

The original contributions presented in this study are included in the article. Further inquiries can be directed to the corresponding author.

## Abbreviations

The following abbreviations are used in this manuscript:

FSS	Frequency-Selective Surface
DCP	Dual Circular Polarization
LHCP	Left-Handed Circular Polarization
RHCP	Right-Handed Circular Polarization
HPBW	Half-Power Beamwidth
AR	Axial Ratio
VSWR	Voltage Standing Wave Ratio
